# The Relationship Between Resting State Quantitative EEG and Daytime Sleepiness Complaints in Parkinson's Disease

**DOI:** 10.1111/jsr.70408

**Published:** 2026-08-16

**Authors:** Thaïna Rosinvil, Ronald B. Postuma, Jean‐Marc Lina, Jacques Montplaisir, Jean‐François Gagnon, Julie Carrier

**Affiliations:** ^1^ Center for Advanced Research in Sleep Medicine Centre de Recherche du CIUSSS‐NÎM Montreal Quebec Canada; ^2^ Department of Psychology Université de Montréal Montreal Quebec Canada; ^3^ Research Center Institut Universitaire de gériatrie de Montréal Montreal Quebec Canada; ^4^ Department of Neurology, Montreal Neurological Institute McGill University Montreal Quebec Canada; ^5^ Department of Electrical Engineering École de Technologie Supérieure Montreal Quebec Canada; ^6^ Centre de Recherches Mathématiques Université de Montréal Montreal Quebec Canada; ^7^ Department of Psychiatry Université de Montréal Montreal Quebec Canada; ^8^ Department of Psychology Université du Québec à Montréal Montreal Quebec Canada

**Keywords:** amplitude‐amplitude coupling (AAC), daytime sleepiness, electroencephalography (EEG), functional connectivity, Parkinson's disease (PD), spectral analyses, weighted phase‐lag‐index (wPLI)

## Abstract

Although many studies have reported the presence of daytime sleepiness in Parkinson's disease (PD), either a weak or no association has been found between self‐reported and current objective measures of daytime sleepiness in this population. Changes in resting state quantitative electroencephalography (qEEG) have been associated with increased subjective sleepiness induced by sleep deprivation in young and older adults. The aim of this study was to investigate the relationship between resting state qEEG and self‐reported daytime sleepiness in adults with PD. Sixty‐two participants with PD underwent polysomnographic recording and clinical assessment. Daytime sleepiness severity was measured with the Epworth Sleepiness Scale (ESS). Resting state EEG was recorded 30 min after awakening. Spectral power and functional connectivity analyses (weighted‐phase lag index, amplitude–amplitude coupling) were performed on artefact‐free resting state EEG in 2 Hz mini bands from 0.5 to 12 Hz. Partial correlations and a hierarchical regression were used to evaluate the relationship between the qEEG metrics and ESS scores. Lower phase synchronisation in the low alpha band (8–10 Hz) within the occipital cortex was the best EEG predictor of higher daytime sleepiness severity in PD. Our results are in line with some findings of previous studies conducted in healthy young participants that reported lower alpha connectivity after sleep deprivation, suggesting disrupted arousal networks may contribute to daytime sleepiness in PD. Lower alpha phase synchronisation within the occipital cortex could serve as a proxy for daytime sleepiness in PD.

**Abbreviations**
AAC: amplitude–amplitude coupling
BAI: beck anxiety inventory
BDI‐II: *Beck Depression Inventory*, Second Edition
CIUSSS‐NÎM: Centre Intégré universitaire de santé et de services sociaux du Nord‐de‐l'Île‐de‐Montréal
DA: dopamine
DOF: dominant occipital frequency
EDS: excessive daytime sleepiness
EEG: electroencephalography
EMG: electromyography
ESS: Epworth Sleepiness Scale
FC: functional connectivity
FDR: false discovery rate
ISI: insomnia severity index
LC: locus coeruleus
LEDD: levodopa equivalent daily dose
MMSE: Mini‐Mental State Examination
MSLT: multiple sleep latency test
MWT: maintenance wakefulness test
PD: Parkinson's disease
PSG: polysomnography
qEEG: quantitative electroencephalography
RBD: REM sleep behaviour disorder
REM: rapid eye movement
ROI: region of interest
UPDRS‐III: unified Parkinson's Disease Rating Scale, Part III
wPLI: weighted phase‐lag index

## 1 Introduction

Excessive daytime sleepiness (EDS) is a critical non‐motor symptom of Parkinson's disease (PD) present in up to 60% of diagnosed individuals and known to worsen clinical outcome as well as the quality of life of both patients and their caregivers (Dodet et al. 2024; Liu et al. 2022). EDS has a complex and multifactorial aetiology in PD (Liu et al. 2022). A principal component analysis conducted recently by our research group found that the interrelationship between a higher dosage of dopamine (DA) agonists, more severe motor symptoms, shorter sleep latency and higher sleep efficiency distinguished individuals who have PD with EDS from those without (Rosinvil et al. 2024).

Although a large number of studies reported the presence of subjective EDS in PD (Liu et al. 2022 for a review), the few that assessed EDS through objective measures in this population showed either a weak association or no significant relationship between these measures and complaint of daytime sleepiness (Arnulf 2005; Bargiotas et al. 2019; Bolitho et al. 2013; Cochen De Cock et al. 2014; Eckhardt et al. 2023; Stauder et al. 2022). Hence, current objective measures to assess daytime sleepiness may not be optimal in PD. The most common tools include the maintenance wakefulness test (MWT; Mitler et al. 2000), multiple sleep latency test (MSLT; Carskadon et al. 1986) and actigraphy. The MWT and MSLT are laboratory‐based objective methods to assess sleep latency under standardised conditions using polysomnography (PSG; e.g., mean MSLT sleep latency < 8 min defined as EDS). Some authors have argued that they lack ecological validity, represent high costs and low discriminative power across diverse clinical populations (Bonnet 2006; Wise 2006). Daytime sleepiness measured by actigraphy identifies sleep episodes that occur outside the main sleep period (i.e., naps) while the patient is at home. While actigraphy is a more ecological measurement (Sadeh 2011), it tends to overestimate sleep and underestimate wake periods and does not identify sleep–wake and wake–sleep transitions as precisely as PSG (Marino et al. 2013; Sadeh 2011). This latter point is problematic, since the main marker of daytime sleepiness in subjective tools is based specifically on these moments of transition from wake to sleep. Furthermore, current objective tools for daytime sleepiness were not designed to measure subtle changes in vigilance state (Bonnet 2006; Sadeh 2011; Wise 2006).

Resting state quantitative electroencephalography (qEEG) is a non‐invasive and cost‐effective technique that provides information on in vivo EEG activities in the brain, which include neural oscillations and neurophysiological mechanisms (Koenig et al. 2020). A general slowing of background resting state qEEG activity in central parietal and occipital cortical areas is commonly reported in PD and is associated with several clinical factors, most prominently cognitive impairment (Bockova and Rektor 2019; Geraedts et al. 2018). However, to the best of our knowledge, no studies have investigated the link between resting state qEEG and daytime sleepiness in PD. Resting state qEEG enables extraction of the spectral power of specific frequencies and functional connectivity (FC), which can provide objective insight into underlying neurophysiological mechanisms of daytime sleepiness. Indeed, studies have indicated that sleep deprivation in young and middle‐aged adults increases resting state theta and low‐alpha (4–9 Hz) spectral power, particularly in frontocentral regions and that the latter is closely linked to subjective sleepiness (Bernardi et al. 2015; Cajochen et al. 1999; Drapeau and Carrier 2004) and behavioural performance decrement (Peng et al. 2025; Robillard et al. 2011). Only a few studies, all performed in young participants (18–40 years), have evaluated the effects of sleep deprivation on FC during resting state EEG. Using different metrics, most reported decreased connectivity in the alpha (7.5–13 Hz) band in different cortical areas (Corsi‐Cabrera et al. 1992; Fasiello et al. 2025; Lian, Xu, Song, Peng, Gong, et al. 2023; Lian, Xu, Song, Peng, Zhang, et al. 2023; Ma et al. 2023; Verweij et al. 2014). Some studies also reported local and global increases in EEG connectivity after sleep deprivation in other specific frequency bands, but results were less consistent (Alonso et al. 2016; Corsi‐Cabrera et al. 1992; Ma et al. 2023; Verweij et al. 2014).

The aim of this study was to evaluate the association between resting state qEEG and subjective daytime sleepiness in adults with PD. We used the Epworth Sleepiness Scale (ESS) as a measure of daytime sleepiness severity. Spectral (absolute and relative power) and FC analyses of phase (weighted phase‐lag index; wPLI) and amplitude synchronisation (amplitude–amplitude coupling; AAC) were used to produce qEEG metrics. We hypothesised that higher theta/low alpha (4–10 Hz) power in frontocentral cortical regions and lower FC in the alpha band (8–12 Hz) would be associated with greater daytime sleepiness complaints in PD.

## 2 Methods

### 2.1 Participants

Individuals between the ages of 45 and 85 years old with idiopathic PD confirmed by a movement disorder specialist were initially recruited for the study (Postuma et al. 2015). Cardinal motor symptoms included bradykinesia and the presence of at least two of the following: rest tremor, rigidity or postural instability (Postuma et al. 2015). They were recruited from the Centre Hospitalier de l'Université de Montréal and the Montreal General Hospital (Montreal, Canada) and invited to participate in a longitudinal study on sleep and cognition in PD. Exclusion criteria were (i) dementia according to the europsychological assessment or the recommendations of the Movement Disorder Society Task Force for PD (Dubois et al. 2007); (ii) a major psychiatric disorder (including bipolar disorder, untreated major depression, schizophrenia) using the criteria of the *Diagnostic and Statistical Manual of Mental Disorders*, Fourth Edition, Text Revision (American Psychiatric Association 2000); (iii) history of stroke or cerebrovascular disease and (iv) PSG date > 6 months since the clinical visit. Individuals with PD continued taking their usual medication during the study. Medication taken to treat motor symptoms of PD was converted to a levodopa equivalent daily dose (LEDD) (Tomlinson et al. 2010). Research protocols were approved by the ethics committee of the Centre Intégré universitaire de santé et de services sociaux du Nord‐de‐l'Île‐de‐Montréal (CIUSSS‐NÎM)—Hôpital du Sacré‐Cœur de Montréal (Montreal, Quebec). All participants gave their informed written consent to participate.

### 2.2 Procedure

Participants underwent a clinical visit at the Centre for Advanced Research in Sleep Medicine of the CIUSSS‐NÎM—Centre de recherche de l'Hôpital du Sacré‐Cœur de Montréal. This included a neurological examination, a complete neuropsychological assessment, sleep and mood questionnaires and a PSG recording. The PSG, performed within the six months following the clinical visit, included a nighttime sleep recording and resting state awake EEG.

#### 2.2.1 Clinical Assessment

Daytime sleepiness severity was measured with the ESS (Johns 1991). Participants also completed the Insomnia Severity Index (ISI) (Bastien et al. 2001). Motor symptom severity was evaluated by a neurologist in the ‘on’ state using the Unified Parkinson's Disease Rating Scale, Part III (UPDRS‐III) (Goetz et al. 2008). The Hoehn and Yahr scale was also used to assess disease severity (Hoehn and Yahr 1967). PD participants underwent a comprehensive neuropsychological assessment measuring five cognitive domains: attention, executive functions, verbal episodic memory and learning, visuospatial and language abilities (Latreille et al. 2015); see Table S3 for the list of neuropsychological tests. Cognitive status was determined by consensus between the neurologist (R.B.P.) and neuropsychologist (J.‐F.G.). Mild cognitive impairment (MCI) diagnosis was based on specific criteria (Litvan et al. 2012) and detailed in a previous article from our group (De Roy et al. 2020). The Mini‐Mental State Examination (MMSE) was also completed to measure global cognitive functioning (Folstein et al. 1975). To assess the severity of depressive and anxiety symptoms, the *Beck Depression Inventory*, *Second Edition* (BDI‐II) and the beck anxiety inventory (BAI) were filled out by the participants (Beck et al. 1988, 1961).

#### 2.2.2 PSG

PSG recordings included EEG (10–20 system, referential montage with linked ears at 10 kΩ resistance), chin electromyogram and bilateral electrooculography. PSG was recorded using a Grass Model 15 amplifier system (gain 10,000; bandpass 0.3–100 Hz). Signals were digitalised at a sampling rate of 256 Hz using commercial software (Harmonie, Stellate System). Sleep stages were identified according to the American Academy of Sleep Medicine criteria (Berry et al. 2012). Oral and nasal airflow plus thoracic and abdominal movements were recorded concurrently with pulse oximetry to record respiratory events during sleep (i.e., apneas and hypopneas). Apneas were defined as an airflow reduction of ≥ 90% lasting ≥ 10s. Hypopneas were defined as an airflow reduction of ≥ 30% lasting ≥ 10s accompanied by either an oxygen desaturation of ≥ 3% or arousal, as recommended (Berry et al. 2012). The aponea hypopnea index (AHI) was calculated by dividing the total number of events (aponeas and hypopneas) by the total sleep time. The diagnostic criteria for REM sleep behaviour disorder (RBD) were (1) REM sleep without atonia, defined as tonic EMG activity > 30% and/or phasic EMG activity > 15% of the total REM sleep time (on 3‐s mini‐epochs within 30‐s REM sleep epochs) and (2) a history of undesirable and potentially harmful behaviours during sleep or observed complex behaviours during REM sleep recorded during the night spent in the laboratory (Cesari et al. 2022; Montplaisir et al. 2010).

#### 2.2.3 Resting State EEG Analyses

In the morning, at least 30 min after awakening from the PSG night recording, a resting state EEG was recorded for 10 min while participants were lying in bed with their eyes closed (wakefulness at rest). Participants were asked to open their eyes at regular intervals to avoid drowsiness.

Resting state qEEG analyses were performed on derivations F3, F4, Fz, C3, C4, Cz, T3, T4, T5, T6, P3, P4, Pz, O1 and O2. Due to eye‐movement‐related artefacts, Fp1 and Fp2 were not considered. Cardiac artefacts from ECG leads were automatically extracted and removed using the signal‐space projection (SSP) algorithm when applicable (Uusitalo and Ilmoniemi 1997). A total of 96 s of EEG sections, each with a minimum duration of 2 s free of eye movements or artefacts, were selected by visual screening per participant, except for two for whom a total of only 48 and 88 s were extracted. The first artefact‐free 96 s of wakefulness were selected. QEEG metrics were first computed on electrodes and further averaged for five regions of interest (ROIs): (1) frontal (F3, F4, Fz), (2) central (C3, C4, Cz), (3) temporal (T3, T4, T5, T6), (4) parietal (P3, P4, Pz) and (5) occipital (O1, O2). Participants needed to have at least 50% of electrodes in each ROI with enough artefact‐free epochs to be included in further statistical analyses (one participant excluded). Since most reported qEEG‐related findings in sleep deprivation studies occur at lower frequencies (< 12 Hz), the present study mainly focused on qEEG correlates for frequencies between 0.5 and 12 Hz.

##### 2.2.3.1 Spectral Analyses

For each electrode, spectral analyses of wakefulness at rest were computed using Matlab (9.6.0) and then averaged for each ROI (frontal, central, parietal, occipital and temporal). Fast Fourier Transformation (cosine tapering) on 2‐s artefact‐free sections yielded a spectral resolution of 0.5 Hz. Absolute and relative spectral power were calculated for low delta (0.5–2 Hz), high delta (2–4 Hz), low theta (4–6 Hz), high theta (6–8 Hz), low alpha (8–10 Hz) and high alpha (10–12 Hz). The dominant occipital frequency (DOF) was defined as the average peak alpha rhythm frequency (8–13 Hz) observed in spectra at O1 and O2 for each participant during wakefulness.

##### 2.2.3.2 FC

Two qEEG FC metrics were used in this study. First, the wPLI (Vinck et al. 2011) was computed between each of the electrodes to assess phase synchronisation. The wPLI is an extension of the phase lag index (PLI; Stam et al. 2007). Since the wPLI is a phase‐based metric, it has the advantage of being independent of amplitude changes and only accounts for the synchrony between the remote oscillations. The wPLI is also blind to volumetric conduction (zero‐lag) across the cortex. Second, the AAC metric was computed between each of the electrodes to assess amplitude synchronisation. In each frequency band, the AAC is the result of the Pearson's correlation coefficient between the Hilbert amplitudes of a pair of narrow band filtered EEG channels (Brazier and Barlow 1956). We evaluated FC within each ROI (intra‐ROI) and in between ROIs (inter‐ROI). To do so, the average value of the wPLI and AAC, respectively, between all electrodes included within a ROI constituted the intra‐ROI FC. To evaluate inter‐ROI couplings, the wPLI and AAC were calculated by averaging the values between each possible pair of ROIs. Consequently, wPLI and AAC in low delta, high delta, low theta, high theta, low alpha and high alpha were computed for 10 pairs of inter‐ROIs (frontal–central, frontal–parietal, frontal–temporal, frontal–occipital, central–parietal, central–occipital, parietal–temporal and parietal–occipital) and five intra‐ROIs (frontal, central, parietal, occipital and temporal).

### 2.3 Statistical Analyses

Statistical analyses were performed using SPSS (version 29) and level of significance was set at 5%. All variables were examined for their mean, standard deviation, skewness and kurtosis. Variables that were not normally distributed (i.e., absolute skew value larger than 2 or an absolute kurtosis larger than 7; Curran et al. 1996) were transformed. To do so, logarithmic transformations were applied for spectral analyses and sleep PSG variables, whereas absolute square‐root transformations were applied for qEEG FC metrics that were not normally distributed.

First, to assess potential confounding variables of daytime sleepiness in PD, Pearson's correlations were performed between ESS score and demographic, clinical and nocturnal sleep PSG variables. PD participants were also divided into two groups based on the presence or absence of EDS, as determined by their score on the ESS (PD with EDS: ESS > 10, PD without EDS: ESS ≤ 10), to assess demographic and clinical variables. To evaluate potential confounding variables such as age, sex, educational level and RBD status, one‐way analyses of variance (ANOVAs) were conducted comparing PD participants with and without EDS on these variables. Only age differed significantly between the two groups (*p* < 0.05). Given the age‐related difference between PD participants with and without EDS, age was controlled for in subsequent analyses. Effect sizes (ESs) were measured using the partial eta squared.

Second, partial correlations were performed to investigate the association between qEEG metrics and ESS when controlling for the significant confounding factors. We adjusted for the false discovery rate (FDR) using the Benjamini–Hochberg procedure on *p*‐values (Benjamini and Hochberg 1995). Corrected *p′‐*values are reported.

Finally, we performed an additional exploratory analysis to identify the qEEG metrics that best predict daytime sleepiness. Since previous studies evaluating the associations between other objective metrics of sleepiness and ESS found ‘weak or no association’ with *r* ranging from −0.28 to −0.33 (Arnulf 2005; Bargiotas et al. 2019; Bolitho et al. 2013; Cochen De Cock et al. 2014), we performed a hierarchical regression including confounding variables in Step 1 and qEEG metrics with a partial correlation absolute value greater than or equal to 0.25 in Step 2. Stepwise selection was performed on the qEEG metrics with an entry *p*‐value threshold of 5% and an exit *p*‐value threshold of 10%.

## 3 Results

For this study, ESS score and resting state EEG were available for an initial group of 84 PD participants. Of these, 22 were excluded: one for dementia, 10 for PSG date > 6 months since the clinical visit and 11 for poor data acquisition (e.g., artefact due to sweating or muscular artefacts present on all derivations). Thus, 62 PD participants were included in subsequent analyses. We found a negative association between age and ESS score, where younger age was associated with higher daytime sleepiness (*r* = −0.31, *p* = 0.016; Table 1). PD participants with EDS were younger than those without EDS (*F* (1,62) = 5.05, *p* = 0.03, ES = 0.08). The other demographic and clinical variables showed no significant associations or group‐differences related to daytime sleepiness in PD. Since age was associated with ESS score, we controlled for this variable in all subsequent analyses. There was no group‐differences nor significant association between PSG variables and ESS score (Table 2).

**TABLE 1:** Sociodemographic and clinical characteristics of PD participants.

Characteristics	All PD (*n* = 62)	PD‐nEDS (*n* = 42)	PD‐EDS (*n* = 20)	Group‐analysis *p* (ES)	Correlation with ESS^b^
Age, years	65.0 ± 8.1	66.6 ± 8.2	61.8 ± 6.9	0.03 (0.08)*	−0.31 (0.016)*
Sex, male *n* (%)	40 (64.5)	27 (64)	13 (65)	1.0	—
Education, years	14.8 ± 3.6	14.9 ± 4.0	14.7 ± 2.5	0.84	−0.00 (0.99)
PD duration (symptoms onset), years	5.8 ± 4.6	5.6 ± 4.5	6.2 ± 4.7	0.63	0.05 (0.72)
PD duration (diagnosis onset), years	4.24 ± 3.5	4.3 ± 3.8	4.1 ± 2.8	0.77	0.02 (0.91)
Hoehn and Yahr stage	2.2 ± 0.8	2.3 ± 0.8	2.2 ± 0.9	0.54	−0.03 (0.82)
UPDRS part III ‘on’ score	20.8 ± 10.1	20.5 ± 10.6	21.5 ± 8.9	0.72	0.10 (0.45)
RBD, *n* (%)	32 (52)	21 (50)	11 (55)	0.79	—
MCI, *n* (%)	32 (52)	21 (50)	11 (55)	0.79	—
MMSE score	28.5 (1.5)	28.5 (1.4)	28.5 (1.7)	0.86	0.02 (0.89)
Medication					
LEDD, mg	603.5 ± 321.1	588.4 ± 317.0	634.6 ± 335.3	0.60	0.19 (0.15)
DA receptor agonist, *n* (%)^a^	21 (34)	12 (29)	9 (45)	0.26	—
DA receptor agonist, LEDD mg	75.1 ± 129.8	52.9 ± 93.8	119.4 ± 176.6	0.06	0.16 (0.23)
Antidepressants, *n* (%)	13 (23)	8 (22)	5 (25)	0.75	—
Anxiolytic, *n* (%)	13 (23)	8 (22)	5 (25)	0.75	—
ISI score	10.8 ± 7.0	10.0 ± 7.2	12.5 ± 6.5	0.20	0.25 (0.06)
BDI‐II score	11.1 ± 7.5	10.6 ± 8.4	12.3 ± 5.4	0.41	0.19 (0.15)
BAI score	11.2 ± 7.7	11.2 ± 8.1	11.3 ± 7.2	1.0	0.09 (0.49)

*Note:* Results are expressed as mean ± standard deviation.

Abbreviations: BAI = Beck Anxiety Inventory; BDI‐II = *Beck Depression Inventory, Second Edition*; DA = dopamine; ES = effect size; ESS = Epworth Sleepiness Scale; ISI = Insomnia Severity Index; LEDD = levodopa equivalent daily dosage; MCI = mild cognitive impairment; MMSE = Mini‐Mental State Examination; PD = Parkinson's disease; PD‐EDS = PD with excessive daytime sleepiness (ESS > 10); PD‐nEDS = PD without EDS (ESS ≤ 10); RBD = rapid eye movement sleep behaviour disorder; UPDRS = unified Parkinson's Disease Rating Scale.

^a^ All on pramipexole.

^b^ *r* (*p*).

* *p* < 0.05.

**TABLE 2:** Polysomnographic variables for PD participants.

Variables	All PD (*n* = 62)	PD‐nEDS (*n* = 42)	PD‐EDS (*n* = 20)	Group‐analysis *p*	Correlation with ESS^b^
Total sleep time, min	342.0 ± 94.7	328.3 ± 103.3	370.9 ± 66.9	0.36	0.06 (0.62)
Sleep latency, min^a^	18.8 ± 24.8	22.0 ± 28.0	12.4 ± 15.2	0.28	−0.12 (0.37)
WASO, min	133.2 ± 76.5	148.6 ± 82.7	100.8 ± 49.2	0.10	−0.17 (0.20)
Sleep efficiency, %	73.8 ± 16.4	70.5 ± 18.0	80.8 ± 9.6	0.08	0.16 (0.23)
Wake, %	26.2 ± 16.5	29.5 ± 18.1	19.2 ± 9.6	0.08	−0.16 (0.23)
N1, %	19.2 ± 9.4	19.8 ± 10.4	18.2 ± 6.6	0.64	−0.25 (0.06)
N2, %	58.3 ± 10.3	58.5 ± 10.9	57.8 ± 8.9	0.72	−0.03 (0.83)
N3, %	6.2 ± 7.6	6.5 ± 7.8	5.4 ± 7.5	0.52	0.14 (0.28)
REM, %	16.3 ± 7.9	15.1 ± 7.8	18.8 ± 7.5	0.10	0.19 (0.14)
REM latency, min	165.7 ± 91.6	176.9 ± 98.1	142.6 ± 73.4	0.28	−0.12 (0.37)
AHI	7.4 ± 8.6	7.4 ± 9.5	7.2 ± 6.7	0.81	−0.05 (0.73)
Mean O_2_ saturation	95.2 ± 1.5	95.1 ± 1.5	95.6 ± 1.5	0.54	0.07 (0.58)
Minimum O_2_ saturation	89.7 ± 3.6	89.4 ± 3.6	90.4 ± 3.5	0.64	0.01 (0.92)
Microarousal index	16.3 ± 6.7	15.7 ± 7.4	17.6 ± 5.2	0.18	−0.09 (0.49)

*Note:* Results are expressed as mean ± standard deviation.

Abbreviations: ESS = Epworth Sleepiness Scale; N1 = non‐rapid eye movement Sleep Stage 1; N2 = non‐rapid eye movement Sleep Stage 2; N3 = non‐rapid eye movement Sleep Stage 3; PD = Parkinson's disease; PD‐EDS = PD with excessive daytime sleepiness (ESS > 10); PD‐nEDS = PD without EDS (ESS ≤ 10); REM = rapid eye movement sleep stage; WASO = wake after sleep onset.

^a^ Log transformed.

^b^ After controlling for age, *r* (*p*‐value).

* *p* < 0.05.

We also evaluated the association between age and clinical factors (see Supporting Information and Table S1). Lower age was associated with higher dopaminergic agonist receptor dosage (*r* = −0.28, *p* = 0.03), higher MMSE score (*r* = −0.50, *p* < 0.001) and lower Hoehn and Yahr stage (*r* = 0.40, *p* = 0.002).

No significant association was found between spectral EEG metrics (absolute and relative spectral power and DOF) and ESS score (see Table S2a). For phase synchronisation, lower wPLI in the low alpha band (8–10 Hz) for the occipital intra‐ROI was associated with higher ESS score (*r* = −0.49, *p′* = 0.003; Figure 1). Similarly, lower wPLI in the low alpha band between five ROIs was associated with higher ESS scores, but the effects did not persist following FDR correction. These included central–parietal (*r* = −0.27, *p* = 0.038), central‐occipital (*r* = −0.27, *p* = 0.036), parietal–temporal (*r* = −0.28, *p* = 0.03), parietal–occipital (*r* = −0.30, *p* = 0.019) and temporal–occipital (*r* = −0.26, *p* = 0.047) regions. No other significant associations were found between phase synchronisation and ESS scores (see Table S2b).

**FIGURE 1:**
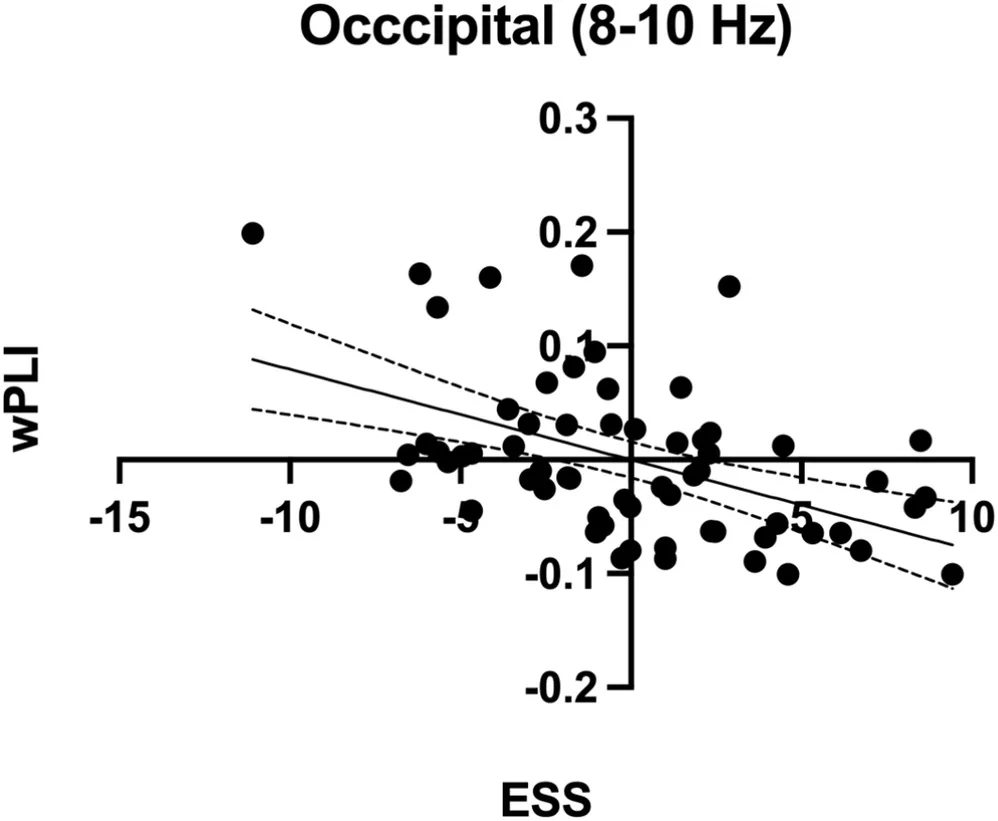
Residual scatterplot of daytime sleepiness and wPLI in occipital intra‐ROI residuals of weighted phase‐lag‐index (wPLI) shown in *y*‐axis and Residuals of Epworth Sleepiness Scale (ESS) shown on the *x*‐axis corrected for age. Full line represents the mean of the regression line. Dash lines represent ±1 standard deviation. ROI = region of interest.

For amplitude synchronisation, no association persisted following FDR‐correction. Nevertheless, uncorrected partial correlations showed that lower AAC for low delta (0.5–2 Hz) between parietal–occipital ROIs (*r* = −0.27, *p* = 0.038) and within parietal ROI (*r* = −0.27, *p* = 0.038) were associated with higher ESS scores. No other significant associations were found between amplitude synchronisation and ESS scores (see Table S2c).

An exploratory two‐step hierarchical multiple regression was then conducted with ESS score as the dependent variable to identify the best predictors within qEEG metrics. Age entered in Step 1 was statistically significant in predicting ESS score and yielded a variance of 9.3% Δ*F* (1, 60) = 6.16, *p* = 0.02 (Table 3). In Step 2, qEEG metrics included wPLI in 8–10 Hz: central–parietal, central–occipital, parietal–temporal, parietal–occipital, temporal–occipital and occipital ROI; wPLI in 10–12 Hz: parietal–occipital and AAC in 0.5–2 Hz: parietal–temporal, parietal–occipital and parietal ROI. Only the wPLI in the occipital area was selected as a significant predictor, adding 21.6% to the total explained variance, Δ*F* (1, 59) = 18.48, *p* < 0.001. Age and the wPLI within the occipital cortex were both significant contributors to ESS score. Overall, the hierarchical regression model accounted for 30.9% of the variance of the ESS score, *F* (2, 59) = 13.22, *p* < 0.0001 (Table 3).

**TABLE 3:** Hierarchical regression analyses predicting ESS score.

Candidate predictors	*B*	*β*	*p*	*R* ^2^	*ΔR* ^2^	*ΔF*	*p*
Step 1: Potential cofounding variables				0.093	0.093	6.16	0.016*
Age	−0.173	−0.305	0.016				
Step 2: qEEG metrics (stepwise selection)^a^				0.309	0.216	18.48	< 0.0001*
Age	−0.182	−0.322	0.004*				
wPLI in occipital area	−30.00	−0.465	< 0.0001*				

Abbreviations: AAC = amplitude–amplitude coupling; DA = dopamine; EDS = excessive daytime sleepiness; ESS = Epworth Sleepiness Scale; PD = Parkinson's disease; ROI = region of interest; wPLI = weighted phase‐lag‐index.

^a^ Stepwise selection (with an entry *p*‐value < 0.05 and an exit *p*‐value < 0.1) included following variables: wPLI in 8–10 Hz: central‐parietal, central‐occipital, parietal–temporal, parietal‐occipital, temporal‐occipital and occipital ROI; wPLI in 10–12 Hz: parietal‐occipital and AAC in 0.5–2 Hz: parietal–temporal, parietal‐occipital and parietal ROI. Only significant predictors of ESS score are included in regression model.

* *p* < 0.05.

## 4 Discussion

This study evaluated the association between resting state qEEG metrics and the severity of self‐reported daytime sleepiness in PD. Our results indicate that lower phase synchronisation in the low alpha band (8–10 Hz) in the occipital area best predicted higher daytime sleepiness complaint in PD. Given the limitations of current objective measures for assessing subjective sleepiness, this result indicates that EEG metrics may be clinically useful, while also offering insights into the neurophysiological underpinnings of daytime sleepiness in PD.

Our results are in line with those of previous studies showing that lower alpha connectivity is associated with increased waking hours in healthy young adults (Alonso et al. 2016; Lian, Xu, Song, Peng, Zhang, et al. 2023). However, contrary to our initial hypothesis that higher theta/low alpha (4–10 Hz) power in frontocentral regions would be associated with greater daytime sleepiness, we found no significant relationship between EEG spectral power and self‐reported daytime sleepiness in our PD cohort. Sleep deprivation studies rely on intra‐individual comparisons, contrasting an EEG at baseline and an EEG following sleep deprivation and thus evaluate changes relative to each individual's unique spectral signature. Our results support the notion that, contrary to spectral EEG spectral power, lower phase synchronisation in the low alpha band is sensitive to inter‐individual differences in subjective sleepiness in adults with PD despite their intra‐individual EEG variations (Tkachenko and Dinges 2018). In addition, although cognitive performance and antiparkinsonian medication intake have been previously associated with EEG FC in PD (Anjum et al. 2024; Bockova and Rektor 2019; Geraedts et al. 2018), they were not correlated with lower phase synchronisation in the occipital area in the present study (Tables S3 and S4). Furthermore, although the correlations between higher ISI scores, lower N1% sleep and ESS scores were not statistically significant, only showing trends (*p* < 0.1), we investigated whether these unexpected trends could have confounded our primary findings. They were not associated with low‐alpha phase synchronisation in the occipital area (Table S4). Taken together, these findings support the notion that lower alpha occipital phase synchronisation is associated with daytime sleepiness in PD.

The neuroanatomical substrates underlying the association between lower phase synchronisation and higher daytime sleepiness complaints in PD remain to be elucidated. Some evidence suggests that white matter integrity may be involved. Myelination of cortico‐cortical axons influences qEEG FC (Nunez and Srinivasan 2014; Nunez et al. 2015), with reduced myelination potentially disrupting the precise timing needed for synchronous neuronal oscillations. However, the precise mechanisms require further investigation, as other factors such as axon density or presence of white matter lesions also influence connectivity (Nunez and Srinivasan 2014; Nunez et al. 2015). Although no study has directly evaluated the relationship between white matter integrity and EEG FC in PD with daytime sleepiness, some studies have reported microstructural white matter changes in PD with EDS, including alterations in the superior and inferior longitudinal fasciculi and superior corona radiata (Ashraf‐Ganjouei et al. 2019; Chondrogiorgi et al. 2016), tracts that project to occipital regions (Janelle et al. 2022). However, in another study, associations between white matter integrity and daytime sleepiness in de novo PD did not survive adjustment for age and sex (Soares et al. 2024). A recent pilot study in early‐stage PD found reduced fronto‐parietal EEG FC without detectable differences in white matter integrity compared to controls (Droby et al. 2022), suggesting that EEG connectivity changes may precede measurable structural damage in the early stages of PD. The relationship between white matter integrity and EEG connectivity in PD with EDS requires further research as it appears complex and stage dependent.

Another complementary hypothesis involves the locus coeruleus (LC), which is the brain's primary source of noradrenaline and modulates cortical arousal states via thalamo‐cortical projections (Dahl et al. 2022). Degeneration of the LC has been reported in PD (Arnulf and Leu‐Semenescu 2009; Doppler et al. 2021; Solopchuk et al. 2018). This noradrenergic depletion has been associated with vigilance impairment in PD (Solopchuk et al. 2018) and there is emerging evidence linking LC integrity and noradrenergic receptor density to altered cortical rhythmic activity in PD and related synucleinopathies (Kemp et al. 2024; Mitterova et al. 2025; Wiesman et al. 2025). However, the association between LC degeneration, EEG changes and EDS in PD requires further investigation. A recent promising study in healthy individuals using in vivo 7 T fMRI has showed that LC tonic activity during wakefulness was associated with REM sleep EEG metrics the following night (Mortazavi et al. 2025). Future multimodal studies assessing daytime sleepiness in PD could incorporate LC‐sensitive imaging methods, such as neuromelanin‐sensitive MRI (Priovoulos et al. 2020), ultra‐high‐field 7 T fMRI (Mortazavi et al. 2025) or PET tracers for noradrenergic transporter density (e.g., 11C‐MeNER; Doppler et al. 2021), alongside EEG connectivity analysis.

Previous studies that assessed EDS in PD using objective measures (e.g., MSLT) reported a weak association or no significant relationship between objective and subjective measures of daytime sleepiness in PD (*r* ranging from −0.28 to −0.33) (Arnulf 2005; Bargiotas et al. 2019; Bolitho et al. 2013; Cochen De Cock et al. 2014; Eckhardt et al. 2023; Stauder et al. 2022). Here, a moderate association was found between lower phase synchronisation in the occipital area in the low‐alpha band and higher daytime sleepiness complaints (*r* = 0.49), explaining 30.9% of the total variance in ESS scores. In our study, younger age was also among the variables predicting daytime sleepiness, accounting for 9.3% of the variance. We sought to further understand this surprising association and found that younger PD participants in our study experienced less severe motor symptoms and were prescribed higher doses of DA receptor agonists, predominantly pramipexole. DA receptor agonists alone can be given as a first line treatment at the beginning of the disease, which could explain the higher dosage found here. Moreover, since pramipexole is known for its sedative effect given a high affinity with D2/D3 receptors (Liu et al. 2022; Rosinvil et al. 2024), it may contribute to the observed association between younger age and higher daytime sleepiness in our PD cohort. Moreover, we cannot rule out the possibility of age‐related differences in the perception of sleepiness among individuals.

Although age and lower phase synchronisation explained a significant portion of variance (30.9%), a substantial portion of EDS variance remained unexplained, thus underscoring the complex interplay of factors influencing daytime sleepiness in PD. Bargiotas et al. (2019) showed that participants with advanced PD underestimated their degree of sleepiness and failed to signal the onset of a sleep episode observed on PSG during the MWT and MSLT, which highlights impaired self‐perception of sleepiness. Their inability to recognise an episode of dosing off to sleep could reflect an altered state of vigilance that has become a new baseline of functioning (Bonnet 2006; Santamaria 2004). This altered state, while not yet objectively quantified in PD, might explain, at least in part, the observed discrepancy between objective and self‐reported daytime sleepiness. Future studies could focus on developing more sophisticated diagnostic approaches for accurately assessing self‐reported daytime sleepiness in individuals with PD.

### 4.1 Limitations of This Study

Given our limited sample of participants with EDS (*n* = 20 with ESS > 10 or 32% of the sample) and to fully exploit the information inherent in the distribution of PD participants' sleepiness complaints in our study, we opted for regression analyses instead of group analyses. This approach avoided the limitations and potential biases of dividing participants into discrete groups while maximising statistical power. It should be noted that this study intentionally excluded participants with dementia, psychiatric comorbidities and a previous history of stroke or cerebrovascular disease. Although these strict selection criteria may affect the size of the resulting sample size of EDS participants, they allow for a deeper understanding of daytime sleepiness in PD without the influence of possible confounding factors, albeit at the cost of reducing the generalizability of our results in the PD population. Hence, the assessment of correlations between cognition, daytime sleepiness and, to some extent, EEG connectivity is limited to mild cognitive changes. Another limitation of our study is the absence of a control group of healthy older participants. This restricts our ability to distinguish between disease‐specific and general age‐ or sleep‐related mechanisms contributing to sleepiness in PD. Furthermore, while our results propose qEEG metrics, particularly low phase synchronisation in the occipital area, as a potential new objective marker, this study did not compare qEEG metrics with conventional objective measures of sleepiness such as MWT or MSLT. Nevertheless, our study demonstrates the relevance of including resting state wake EEG in research or clinical paradigms using MWT or MSLT and implementing connectivity analyses. Such an approach would provide a more comprehensive understanding of the neurophysiological underpinnings of daytime sleepiness in PD.

## 5 Conclusion

This study identifies a novel association between lower phase synchronisation in the low‐alpha band within the occipital cortex and higher daytime sleepiness complaints in PD. This finding is of particular interest given the limitations of existing objective measures for assessing daytime sleepiness in this population. Lower alpha phase synchronisation may reflect disrupted neural networks involved in arousal regulation or vigilance perception in PD. Future research incorporating larger samples, longitudinal assessment to test for intra‐individual differences and multimodal imaging is warranted to validate the potential of qEEG phase synchronisation as a sensitive biomarker for sleepiness in PD and to expand knowledge of the neuronal network involved.

## Author Contributions

**Jean‐Marc Lina:** formal analysis, writing – review and editing. **Thaïna Rosinvil:** writing – original draft, writing – review and editing, formal analysis. **Ronald B. Postuma:** conceptualization, methodology, investigation, writing – review and editing. **Jacques Montplaisir:** conceptualization, methodology, writing – review and editing.

## Funding

This work was supported by Fonds de Recherche du Québec—Santé, Canadian Institutes of Health Research and Canada Research Chairs.

## Conflicts of Interest

T.R. received PhD scholarships from the Fonds de Recherche du Québec—Santé (FRQ‐S) and the Canadian Institutes of Health Research (CIHR). R.B.P. received grants from the CIHR and the National Institutes of Health (NIH)/National Institute on Aging (NIA). He received personal compensation for travel, speaker fees and consultation from Biotie, Biogen, Boehringer‐Ingelheim, Roche and Teva Neurosciences, outside the submitted work. J.M. received personal compensation for consultancy services from Takeda, Merck, Paldin and Eisai outside the submitted work. J.M. received grants from the CIHR. He holds a Canada Research Chair in Sleep Medicine. J.C. received grants from the CIHR. She received personal compensation for consultancy services from Eisai outside the submitted work. J.‐F.G. received grants from the CIHR, the FRQ‐S and the NIH/NIA. He holds a Canada Research Chair in Cognitive Decline in Pathological Aging. The other author declares no conflicts of interest.

## Supporting information


**Table S1:** Clinical characteristics of PD participants in association with age.
**Table S2:** (a) Spectral EEG metrics (absolute and relative spectral power and DOF) and (b) phase synchronisation.
**Table S3:** Cognitive performance of PD participants in relation to wPLI in occipital area.
**Table S4:** Controlling for additional confounding variables for wPLI in occipital area.

## Data Availability

The data that support the findings of this study are available on request from the corresponding author. The data are not publicly available due to privacy or ethical restrictions.
