# Supplementary material for: The Relationship Between Resting State Quantitative EEG and Daytime Sleepiness Complaints in Parkinson's Disease
Source: J Sleep Res. 2026 Aug 16;35(5):e70408. doi: 10.1111/jsr.70408 (PMC13619040; doi:10.1111/jsr.70408)
Supplement: Supplementary file 1 [file JSR-35-e70408-s001.docx]

**SUPPLEMENTARY MATERIAL**

**Supplementary Results**

To better understand the association of age with daytime sleepiness we performed Person’s correlation between age and clinical factors (See Supplementary Table 2). Younger age was associated with higher intake of dopaminergic agonist receptor dose (*r* = -.28, *p* = .03), better global cognition (MMSE score, *r* = -.50, *p* < .001), and less severe disease symptoms (Hoeh and Yahr stage, *r* = .40, *p* = .002).

**Supplementary Table 1.**

*Clinical characteristics of PD participants in association with age*

| Characteristics | All PD  (n=62) | Age  r (*p*-value) |
| --- | --- | --- |
| PD duration (symptoms onset), y | 5.8 ± 4.6 | -.14 (.28) |
| PD duration (diagnosis onset), y | 4.24 ± 3.5 | -.03 (.85) |
| Hoehn and Yahr stage | 2.2 ± 0.8 | .40 (.002)* |
| UPDRS part III ‘on’ score | 20.8 ± 10.1 | .13 (.30) |
| MMSE score | 28.5 ± 1.5 | -.50 (<.001)* |
| Medication |  |  |
| LEDD, mg | 603.5 ± 321.1 | -.04 (.77) |
| DA receptor agonist, LEDD mg | 75.1 ± 129.8 | -.28 (.03)* |
| ISI score | 10.8 ± 7.0 | -.18 (.18) |
| BDI-II score | 11.1 ± 7.5 | .16 (.22) |
| BAI score | 11.2 ± 7.7 | .18 (.16) |

Results are expressed as mean ± standard deviation.

PD=Parkinson’s disease. ESS=Epworth Sleepiness Scale. DA=Dopamine. UPDRS=Unified Parkinson’s Disease Rating Scale. LEDD=Levodopa Equivalent Daily Dosage. MMSE=Mini-Mental State Examination. ISI=Insomnia Severity Index. BDI-II=Beck Depression Inventory second edition. BAI=Beck Anxiety Inventory.

^†^All on pramipexole.*Statistical significance, *p* < .05

**Supplementary Table 2.**

a. ***Spectral EEG metrics (absolute and relative spectral power, and DOF)***

|  | **Absolute Spectral Power** | | | | |  | **Relative Spectral Power** | | | | |  | **DOF  (10-13 Hz)** |
| --- | --- | --- | --- | --- | --- | --- | --- | --- | --- | --- | --- | --- | --- |
|  | Frontal | Central | Parietal | Temporal | Occipital |  | Frontal | Central | Parietal | Temporal | Occipital |  |  |
|  | **0.5 - 2 Hz** | | | | |  | **0.5 - 2 Hz** | | | | |  |  |
| r | -.07 | -.04 | -.02 | -.02 | -.02 |  | -.04 | .05 | .12 | .08 | .14 |  | -.13 |
| (p-value) | .57 | .74 | .87 | .91 | .86 |  | .74 | .69 | .38 | .53 | .29 |  | .31 |
|  | **2 - 4 Hz** | | | | |  | **2 - 4 Hz** | | | | |  |  |
| r | -.03 | -.05 | -.05 | -.06 | -.07 |  | .02 | .10 | .19 | .10 | .14 |  |  |
| (p-value) | .83 | .73 | .68 | .67 | .57 |  | .86 | .44 | .14 | .43 | .27 |  |  |
|  | **4 - 6 Hz** | | | | |  | **4 - 6 Hz** | | | | |  |  |
| r | -.05 | -.10 | -.10 | -.06 | -.05 |  | -.04 | -.01 | .05 | .02 | .10 |  |  |
| (p-value) | .69 | .46 | .45 | .67 | .70 |  | .74 | .95 | .68 | .91 | .45 |  |  |
|  | **6 - 8 Hz** | | | | |  | **6 - 8 Hz** | | | | |  |  |
| r | -.02 | -.04 | -.09 | -.02 | -.04 |  | .13 | .06 | .01 | .05 | .06 |  |  |
| (p-value) | .86 | .75 | .51 | .87 | .79 |  | .32 | .62 | .97 | .71 | .65 |  |  |
|  | **8 - 10 Hz** | | | | |  | **8 - 10 Hz** | | | | |  |  |
| r | -.13 | -.08 | -.11 | -.09 | -.12 |  | -.06 | .03 | -.03 | -.07 | -.10 |  |  |
| (p-value) | .34 | .53 | .42 | .50 | .37 |  | .66 | .85 | .83 | .61 | .44 |  |  |
|  | **10 - 12 Hz** | | | | |  | **10 - 12 Hz** | | | | |  |  |
| r | -.08 | -.15 | -.18 | -.08 | -.14 |  | -.06 | -.16 | -.14 | -.07 | -.11 |  |  |
| (p-value) | .54 | .25 | .17 | .53 | .28 |  | .64 | .21 | .30 | .62 | .39 |  |  |

*Statistical significance, *p* < .05. Uncorrected *p* – value reported. EEG=electroencephalography; DOF=Dominant Occipital Frequency.

b. ***Phase synchronization***

|  | **wPLI Intra-ROI** | | | | | |  | | **wPLI Inter-ROIs** | | | | | | | | | | | | | | | | | | | |  |
| --- | --- | --- | --- | --- | --- | --- | --- | --- | --- | --- | --- | --- | --- | --- | --- | --- | --- | --- | --- | --- | --- | --- | --- | --- | --- | --- | --- | --- | --- |
|  | Frontal | Central | Parietal | Temporal | | Occipital |  | | Frontal-Central | | Frontal-Parietal | | Frontal-Temporal | | Frontal-Occipital | | Central-Parietal | | Central-Temporal | | Central-Occipital | | Parietal-Temporal | | Parietal-Occipital | | Temporal-Occipital | |  |
|  | **0.5 - 2 Hz** | | | | | |  | | **0.5 - 2Hz** | | | | | | | | | | | | | | | | | | | |  |
| r | .19 | -.06 | .03 | -.13 | | -.07 |  | | .06 | | -.17 | | -.17 | | -.15 | | .01 | | .02 | | -.05 | | .00 | | -.09 | | -.03 | |  |
| (p-value) | (.15) | (.65) | (.82) | (.31) | | (.61) |  | | (.67) | | (.20) | | (.19) | | (.24) | | (.91) | | (.86) | | (.73) | | (1.00) | | (.47) | | (.82) | |  |
|  | **2 - 4 Hz** | | | | | |  | | **2 - 4 Hz** | | | | | | | | | | | | | | | | | | | |  |
| r | -.08 | -.06 | .12 | .14 | | .05 |  | | -.21 | | -.18 | | .02 | | -.17 | | .11 | | .02 | | .02 | | .00 | | .16 | | -.03 | |  |
| (p-value) | (.57) | (.63) | (.38) | (.29) | | (.72) |  | | (.11) | | (.17) | | (.89) | | (.19) | | (.40) | | (.89) | | (.86) | | (.99) | | (.23) | | (.82) | |  |
|  | **4 - 6 Hz** | | | | | |  | | **4 - 6 Hz** | | | | | | | | | | | | | | | | | | | |  |
| r | -.01 | .12 | -.15 | .04 | | .02 |  | | .03 | | .00 | | .10 | | .21 | | -.08 | | .14 | | .13 | | .14 | | .10 | | .23 | |  |
| (p-value) | (.92) | (.37) | (.27) | (.79) | | (.87) |  | | (.84) | | (.99) | | (.42) | | (.10) | | (.54) | | (.27) | | (.30) | | (.29) | | (.46) | | (.08) | |  |
|  | **6 - 8 Hz** | | | | | |  | | **6 - 8 Hz** | | | | | | | | | | | | | | | | | | | |  |
| r | .20 | .03 | .06 | .08 | | .00 |  | | -.01 | | -.05 | | -.02 | | -.13 | | .00 | | .04 | | -.16 | | .03 | | -.14 | | -.03 | |  |
| (p-value) | (.13) | (.83) | (.68) | (.52) | | (1.00) |  | | (.96) | | (.70) | | (.86) | | (.31) | | (1.00) | | (.79) | | (.22) | | (.83) | | (.28) | | (.85) | |  |
|  | **8 - 10 Hz** | | | | | |  | | **8 - 10 Hz** | | | | | | | | | | | | | | | | | | | |  |
| r | -.14 | -.09 | -.24 | -.20 | **-.49** | | |  | | -.22 | | -.24 | | -.18 | | -.24 | | **-.27** | | -.19 | | **-.27** | | **-.28** | | **-.30** | | **-.26** | |
| (p-value) | (.27) | (.48) | (.06) | (.13) | **(<.0001) *** | | |  | | (.09) | | (.07) | | (.17) | | (.06) | | **(.04) *** | | (.14) | | **(.04) *** | | **(.03) *** | | **(.02) *** | | **(.05) *** | |
|  | **10 - 12 Hz** | | | | | |  | | **10 - 12 Hz** | | | | | | | | | | | | | | | | | | | |  |
| r | -.10 | -.03 | -.18 | -.11 | | -.25 |  | | -.15 | | -.17 | | -.11 | | -.13 | | -.16 | | -.18 | | -.21 | | -.24 | | -.25 | | -.12 | |  |
| (p-value) | (.43) | (.84) | (.17) | (.41) | | (.06) |  | | (.25) | | (.19) | | (.41) | | (.34) | | (.21) | | (.16) | | (.11) | | (.07) | | (.05) | | (.36) | |  |

wPLI = weighted Phase Lag Index. ROI=Region of interest.

*Statistical significance, *p* < .05. Uncorrected *p* – value reported.

c. ***Amplitude synchronization***

|  | **AAC Intra-ROI** | | | | |  | **AAC Inter-ROIs** | | | | | | | | | |
| --- | --- | --- | --- | --- | --- | --- | --- | --- | --- | --- | --- | --- | --- | --- | --- | --- |
|  | Frontal | Central | Parietal | Temporal | Occipital |  | Frontal-Central | Frontal-Parietal | Frontal-Temporal | Frontal-Occipital | Central-Parietal | Central-Temporal | Central-Occipital | Parietal-Temporal | Parietal-Occipital | Temporal-Occipital |
|  | **0.5 - 2 Hz** | | | | |  | **0.5 - 2Hz** | | | | | | | | | |
| r | .04 | -.07 | **-.27** | -.16 | -.06 |  | -.06 | -.17 | -.05 | -.02 | -.23 | -.19 | -.21 | **-.26** | **-.27** | -.24 |
| (p-value) | .74 | .58 | **.04*** | .21 | .63 |  | .62 | .19 | .72 | .90 | .07 | .14 | .11 | **.04*** | **.04*** | .07 |
|  | **2 - 4 Hz** | | | | |  | **2 - 4 Hz** | | | | | | | | | |
| r | .09 | -.02 | -.20 | -.08 | -.08 |  | .09 | .02 | .10 | -.04 | -.08 | -.11 | -.16 | -.23 | -.21 | -.21 |
| (p-value) | .49 | .88 | .12 | .56 | .53 |  | .48 | .89 | .44 | .75 | .56 | .41 | .23 | .08 | .10 | .10 |
|  | **4 - 6 Hz** | | | | |  | **4 - 6 Hz** | | | | | | | | | |
| r | .07 | -.01 | -.17 | .00 | -.04 |  | .02 | -.08 | .06 | .02 | -.15 | -.12 | -.14 | -.23 | -.15 | -.13 |
| (p-value) | .58 | .93 | .20 | 1.00 | .79 |  | .86 | .52 | .62 | .90 | .26 | .35 | .28 | .08 | .24 | .31 |
|  | **6 - 8 Hz** | | | | |  | **6 - 8 Hz** | | | | | | | | | |
| r | -.05 | -.05 | -.19 | -.05 | -.14 |  | -.02 | -.05 | .10 | -.03 | -.14 | -.10 | -.15 | -.17 | -.20 | -.15 |
| (p-value) | .71 | .70 | .14 | .71 | .29 |  | .88 | .71 | .45 | .84 | .27 | .43 | .26 | .19 | .12 | .26 |
|  | **8 - 10 Hz** | | | | |  | **8 - 10 Hz** | | | | | | | | | |
| r | -.10 | .06 | -.05 | .00 | -.02 |  | .04 | .03 | .17 | .01 | .03 | .10 | .04 | -.05 | -.07 | -.08 |
| (p-value) | .43 | .66 | .70 | .98 | .86 |  | .75 | .82 | .18 | .97 | .79 | .47 | .76 | .71 | .60 | .53 |
|  | **10 - 12 Hz** | | | | |  | **10 - 12 Hz** | | | | | | | | | |
| r | -.07 | .07 | -.13 | .05 | -.10 |  | .17 | .23 | .25 | .15 | .06 | -.01 | .00 | -.16 | -.15 | -.18 |
| (p-value) | .59 | .60 | .32 | .71 | .45 |  | .19 | .07 | .05 | .24 | .65 | .93 | .98 | .21 | .24 | .17 |

AAC=Amplitude-Amplitude Coupling. ROI=Region of interest.

*Statistical significance, *p* < .05. Uncorrected *p* – value reported.

**Supplementary Table 3.**

*Cognitive performance of PD participants in relation to wPLI in occipital area.*

| **Cognitive performance** | **All PD**  **(n=62)** | **Correlation with wPLI in occipital area^1^** |
| --- | --- | --- |
| **Attention** |  |  |
| *Bell’s Test (omission)* | -.11 ± 1.0 | .17 (.18) |
| *WAIS-IV: Digit Span (forward)* | .30 ± .83 | -.22 (.09) |
| *Stroop Color Word-Test* |  |  |
| *Condition 3 – Condition 2* | -.81 ± 1.1 | .05 (.72) |
| *Condition 3 – Condition 1* | -.94 ± 1.2 | .04 (.74) |
| *Trail Making Test A* | -1.3 ± 1.5 | -.15 (.25) |
|  |  |  |
| **Executive function** |  |  |
| *WAIS-IV: Digit Span (backward)* | .01 ± .91 | -.20 (.12) |
| *Stroop Color Word-Test* |  |  |
| *Condition 4 – Condition 3* | .13 ± 1.2 | -.02 (.89) |
| *Trail Making Test B* | -1.6 ± 1.6 | -.02 (.87) |
| *Trail Making test B-A* | -.96 ± 1.6 | -.06 (.66) |
| *Verbal fluency* |  |  |
| *Phonologic* | -.40 ± 1.0 | -.03 (.85) |
| *Semantic* | -1.0 ± .86 | .07 (.61) |
| **Visuospatial abilities** |  |  |
| *Rey complex figure: copy* | -.45 ± 1.2 | -.01 (.95) |
| *WAIS-IV: Block design* | -.19 ± 1.0 | .09 (.52) |
| **Language** |  |  |
| *MMSE (score /8)^#^* | 7.9 ± .4 | .00 (.98) |
| **Learning and Memory** |  |  |
| *RAVLT Total trials 1 to 5* | .01 ± 1.3 | .10 (.44) |
| *RAVLT Immediate Recall* | -.27 ± 1.1 | -.03 (.84) |
| *RAVLT Delayed Recall* | .12 ± 1.2 | -.04 (.75) |

Results are expressed as the mean of z-score ± standard deviation.

PD=Parkinson’s disease. wPLI=weighted Phase-Lag Index. WAIS-IV=Wechsler Adult Intelligence Scale, Fourth Edition. MMSE=Mini-Mental State Examination. RAVLT=Rey Auditory Verbal Learning Test.

^#^Raw score.

^1^*r* (p-value) after controlling for age.

*Statistical significance, *p* < .05

**Supplementary Table 4.**

*Controlling for additional confounding variables for wPLI in occipital area*

| Characteristics | All PD  (n=62) | Correlations with wPLI in occipital area^1^ |
| --- | --- | --- |
| ISI | 10.8 ± 7.0 | -.11 (.40) |
| N1, % | 19.2 ± 9.4 | .15 (.24) |
| Medication |  |  |
| LEDD, mg | 603.5 ± 321.1 | -.15 (.26) |
| DA receptor agonist, LEDD mg | 75.1 ± 129.8 | -.08 (.54) |

Results are expressed as mean ± standard deviation.

wPLI=weighted Phase Lag Index. ISI=Insomnia Severity Index. N1=Non-rapid eye movement Sleep Stage 1. DA=Dopamine. LEDD=Levodopa Equivalent Daily Dosage.

^†^Motor status, DA agonist, sleep latency and efficiency component score is comprised of shorter sleep latency, greater sleep efficiency, higher dosage of DA agonist dosage (expressed in LEDD) and more severe motor symptoms (expressed with the Unified Parkinson’s Disease Rating Scale (UPDRS)).

^1^*r* (p-value) after controlling for age.

*Statistical significance, *p* < .05
